# Nursing Students’ Perceptions of Smartphone Use in the Clinical Care and Safety of Hospitalised Patients

**DOI:** 10.3390/ijerph18031307

**Published:** 2021-02-01

**Authors:** Vanesa Gutiérrez-Puertas, Lorena Gutiérrez-Puertas, Gabriel Aguilera-Manrique, Mᵃ Carmen Rodríguez-García, Verónica V. Márquez-Hernández

**Affiliations:** 1 Department of Nursing, Physiotherapy and Medicine, Faculty of Health Sciences, University of Almeria Sacramento S/N, en La Cañada de San Urbano, CP 04120 Almería, Spain; vgp919@ual.es (V.G.-P.); gaguiler@ual.es (G.A.-M.); mrg451@ual.es (M.C.R.-G.); vmh380@ual.es (V.V.M.-H.); 2 Research Group of Health Sciences, CP 04120 Almería, Spain

**Keywords:** clinical setting, interpersonal communication, nursing students, patient care, patient safety, qualitative research, smartphone

## Abstract

Smartphones have become an indispensable item for nursing students. The use of these devices in the clinical setting could have various effects on the clinical work of nursing students. This study was to explore nursing students’ perceptions of their lived experiences of smartphone use in the clinical setting, in regard to patient safety. A descriptive phenomenological study was carried out. A total of 24 nursing students from a university in the southeast of Spain participated in this study. There were 10 in-depth interviews and two focal groups from January to May 2020. The data analysis was performed using ATLAS.TI software to identify the emergent topics. The COREQ Checklist was used to prepare the manuscript. Three principal topics were identified that illustrated the nursing students’ experiences and perceptions of smartphone use in the clinical setting (1): Using smartphones in the clinical setting as a personal resource, (2) smartphones as a support mechanism for making clinical decisions, (3) impact of smartphones on patient care. The nursing students perceived smartphones as a support mechanism for making clinical decisions and for patient care. Smartphone use during clinical practicums may influence the quality of patient-centred communication and threaten clinical safety. The results of this study provide knowledge on the use of smartphones by nursing students in the clinical setting, which could help to establish measures that guarantee adequate patient care and responsible use of these devices.

## 1. Introduction

Smartphones have become vital to our lives due to their many features, such as their size, portability, and access to the internet at any time and place [1]. Smartphones have changed human interaction as they provide the opportunity to communicate and receive feedback, as well as to access, store, save, and share information quickly [2].

Some features of smartphones have become a useful resource for nursing students in the clinical setting [3]. Smartphones make it easier to calculate drug doses and access information related to health and clinical tools [4,5]. For these reasons, smartphones could have significant potential to support nursing students’ clinical decision-making, as well as to improve the care provided to patients [6]. However, there is a lack of clarity about the quality and credibility of the resources that nursing students use on their smartphones [7]. In addition, smartphones encourage communication between peers, preceptors or nurse educators, enabling them to share information, experience or advice, constituting a part of their training [8]. Furthermore, smartphone use can help to improve self-efficacy [9], clinical competencies, promote clinical efficiency and consequently minimise the stress of nursing students during their clinical practicums [5]. 

Nonetheless, in recent years, the use of smartphones for personal use has increased in the clinical setting, with social networks being the main activity [10]. The inappropriate use of social networks in a clinical setting may impair the work and learning environment and negatively interfere with patient safety [11]. Specifically, disruptions caused by smartphones have a negative impact in the clinical setting because they distract the nursing students’ attention, negatively affecting the performance of the activity they are doing [12]. This can cause them to overlook possible complications during techniques or procedures [13]. A recent study links the telepressure of private life with cognitive failure in nurses’ clinical settings, due to having their smartphone nearby (usually, in their pocket), regardless of the notification settings of the phone [14]. Furthermore, the misuse of smartphones in the clinical setting can compromise confidentiality and privacy of patients and lead to a lack of professionalism [15,16]. In addition, the use of smartphones may increase the risk of the transmission of bacterial agents to the patients and therefore increase the risk of hospital infections [17].

Regarding problematic smartphone use among nursing professionals, the use of smartphones has been associated with a decrease in self-management skills and lower performance in the clinical setting [18], as well as the increase in fatigue, which may lead to poorer work performance and clinical errors, and thus, interfere with patient safety [19]. 

Various investigations address the problematic use of smartphones among nursing students, indicating high levels of addiction to their smartphones [20,21] and nomophobia “no mobile phobia” [22]. Specifically, the problematic use of smartphones among nursing students is associated with lower quality of sleep [23], lower self-esteem and an increase in depression [24], lower perceived social support [25], and psychological distress, negatively affecting nursing students’ learning in the clinical setting [26]. Additionally, in the clinical setting, problematic smartphone use among nursing students is associated with perceived work overload [27], greater work fatigue [28], and a negative impact on clinical decision-making [29]. Moreover, it has been shown to negatively influence the communication skills of nursing students with patients and professionals [20]. Likewise, the problematic use of smartphones increases the risk of distraction during the clinical practicums and could therefore be a threat to the patient’s health [30,31]. 

Based on the above, professionals and nursing students are essential to ensure the adequate care and clinical safety of patients. Similarly, smartphones are becoming an indispensable item for nursing students in the clinical setting [8]. However, there is little evidence on the types of smartphone use and the impact they could have in the clinical setting. Along the same line, no studies have been carried out that explore nursing students’ experiences and perceptions of smartphone use in the clinical setting and how it could interfere with patient safety. For this reason, the objective of this study was to explore nursing students’ perceptions of their lived experiences of smartphone use in the clinical setting, in regard to patient safety.

## 2. Materials and Methods

### 2.1. Design

A descriptive phenomenological approach was used to guide to enable a rich understanding of the participants´ life experience. Phenomenology was created to explore the meanings of lived experiences, to be directly open to phenomena and to be able to perceive “the thing” themselves [32]. This means that phenomenological researchers are interested in describing individuals´ experiences in the way they experience them [33]. This study followed the consolidated criteria for reporting qualitative research, the (COREQ) 32-item checklist [34].

### 2.2. Participants

The nursing students were selected through a sample technique for intentional convenience, in order to obtain relevant information to address the objective of this study. The inclusion criterion established were: (a) have and use a smartphone; (b) be enrolled in practicum subjects and have taken at least one practicum subject. Being an exchange student was established as exclusion criteria. 

### 2.3. Data Collection

Once permission was obtained from the Institutional Review Board, the nursing students were contacted by email to invite them to participate in the study. A date and time for the focus group (FG) and the in-depth interviews (IDI) was agreed upon by the nursing students interested in participating. A total of 24 nursing students agreed to participate. Data collection included two FGs consisting of seven students each. The FGs allowed the participants to spontaneously express their perceptions and experiences and reflect on them, creating an exchange of ideas [16], and the 10 IDIs were used to delve into the topics that arose during the FGs. The FGs lasted about 40 min each, and the IDIs lasted 18–30 min. The FGs and IDIs were carried out in a faculty laboratory. The research team developed a semi-structured interview guide, based on literature review, and used it to carry out each of the interviews (Table 1). 

To create a feeling of trust, all of the IDIs and FGs were carried out by a nurse who was part of the research team. Previously, the nurse was given instructions on how to conduct the interviews to feel as natural as possible, using the question script for support rather than strictly following it. The sociodemographic characteristics of the participants were collected. The data collection was carried out until the data were saturated. Data collection took place from January to May 2020.

### 2.4. Data Analysis

The IDIs and FGs were audio recorded with each participant’s permission and were then transcribed verbatim. A hermeneutical unit was created and analysed using ATLAS.TI version 8.0. For data analysis, to ensure the reliability and validity of the results the research followed the Colaizzi [35] methods of descriptive phenomenological data analysis: (1) Completed transcript of the interview and understood the participants´ lived experiences; (2) Significant sentences were scrutinized to meaningful statement; (3) Meaningful statements were extracted to meaningful units; (4) Meaningful statements were classified into subthemes and themes; (5) Themes and subthemes were integrated into a comprehensive description of the participants´ lived experience; (6) The basis structure of the participants´ lived experienced was described; (7) All interviewees analyzed the findings for verification of the accuracy of the transcripts and resemblance of their experiences. The most relevant citations were selected to be included in the study. 

### 2.5. Ethical Considerations

The study was approved by the Research Committee of the Department of Nursing, Physical Therapy and Medicine at the University of Almeria (EFM-63/20). The participants were informed about the objectives of the study and its voluntary nature, as well as the anonymous and confidential treatment of their data. In order to guarantee that the data would be anonymous and confidential, all of the interviews were assigned a code. The participants were told that they could leave the study at any time. Before starting the research, they were required to sign an informed consent form. Additionally, the participants were asked permission to record the conversations and were given access to the study results. The guidelines established by the Declaration of Helsinki were followed at all times.

### 2.6. Rigor

To ensure the reliability and rigor of the qualitative data, it was triangulated among three researchers (LGP, VMH, VGP) who analysed the data separately, discussing any differences until reaching a consensus in the selection of topics and subtopics to increase reliability. An independent investigator (GAM) read the transcripts of the IDIs and FGs to confirm their agreement with the obtained findings (topics and subtopics), verifying that all of the participants’ perspectives were considered. Recordings, data analysis, and interviews were kept to ensure reliability. In order to ensure confirmability, the participants verified the transcription and data analysis. 

## 3. Results

The total study sample consisted of 24 nursing students of which 79.2% (n = 19) were women and 20.8% (n = 5) were men. A total of 10 students participated in individual interviews and 14 participated in FGs, 7 in each of the FGs. The average age was 21.37 (SD = 3.68; range = 19–32). The sociodemographic characteristics of the participants can be seen in Table 2. 

The three main topics, subtopics and meaning units that emerged from the analysis are presented in Table 3.

### 3.1. Smartphone Use as a Personal Resource in the Clinical Setting

One of the main topics that emerged from the interviews was the importance that participants placed on using smartphones in the clinical setting for personal matters. Participants felt the need to release their emotions and disconnect from their professional work due to the experiences they had to face during the workday. 

#### 3.1.1. Smartphones as a Means of Personal Gratification

The nursing students perceived smartphones as an indispensable item in their lives, essential to satisfy the need to be connected that they felt during their clinical practicums. Smartphone separation provoked anxiety and recurrent thoughts in the nursing students due to the fear of being disconnected, coming to realise their smartphone addiction.

“For me, forgetting my smartphone makes me feel like I’m missing something, I feel a bit of anxiety thinking about who could have tried to talk to me or message me.”

(DIS3)

“I’m always looking at my smartphone, I always have it on me. Nowadays, we’re addicted to our smartphones at work and really, everywhere.”

(FGS1)

The nursing students pointed out that their work hours were long and they often faced stressful situations. For this reason, they needed to use their smartphones for personal reasons to escape the clinical setting and alleviate their emotional burden. This led nursing students to perceive the smartphone as an escape from their clinical work.

“Work shifts are really long and sometimes it’s necessary. I use it for other things than work… I need it.”

(D1S1)

“(When you use your smartphone) you disconnect from wherever you are… if you’ve had a stressful work shift… well, you can escape a bit, and feel as if you weren’t in the hospital, and it helps.”

(FGS2)

On the other hand, the participants argued that they were forced to use their smartphones in the nurses’ lounge because the other professionals did. Using smartphones in the clinical setting was perceived as an obstacle for social relationships between colleagues, causing feelings of emptiness and isolation.

“In the (nurses’) lounge, no one pays any attention to you, you don’t know who to talk to so you take out your smartphone because everyone else is using theirs. In the end, it’s as if you don’t have workmates, you mind your own business…”

(DIS10)

#### 3.1.2. Failure of Professional Competence

Smartphone use in the clinical setting could affect the responsibilities of nursing professionals, interfering with their daily routine by delaying their principal duties, repeating something twice, or carrying out unnecessary tests. In addition, the participants reported that the use of smartphones on certain occasions could lead the professionals to free themselves from their clinical work, delegating their activities to nursing students, without ensuring their proper performance or quality.

“Sometimes you’re so focused on your WhatsApp conversations that you don’t hear your workmate, so you don’t you realise that the patient has already had their tests done or that the doctor has already gone by the room… and then you go to do it…”

(DIS3)

“They send the trainee, yeah, let him (the nursing student) do it. Meanwhile, (the nursing professional) is on Facebook… without a worry in the world, you really see that happen, it’s the reality.”

(FGS1)

The nursing students commented that the preceptors on some occasions did not pay them adequate attention, failing to fulfill their teaching duty. This generated feelings of inferiority in the nursing students and negative feelings towards their tutor. Students indicated the disinterest of preceptors negatively interfered with their clinical training and team relationships.

“They (the preceptors) start using their smartphone, you ask them something and they don’t respond, they ignore you… their smartphone seems to be more important than you, and it’s not a good feeling… it’s not the same.”

(DIS5)

### 3.2. Smartphone as a Support Mechanism for Making Clinical Decisions 

This category focused on the nursing students’ experiences regarding smartphone use during clinical practicums. The nursing students used their smartphones in various clinical situations in order to resolve questions on their own that arose during the training process. In addition, the participants perceived their smartphones as a support mechanism for making decisions, improving the development of their clinical work. The topic contains two subtopics:

#### 3.2.1. Support Mechanism for Care

Smartphones were perceived by the nursing students as a useful tool to resolve doubts that arose during their practicums, allowing them to reinforce their clinical knowledge and skills to improve the quality of care they provided.

“I’ve used it to look up medications that I wasn’t exactly familiar with or didn’t know how to administer them, to calculate doses, or things like that. I’ve also used it to look up care methods or watch videos of the procedures that I’m going to do… it has gotten me out of difficult situations.”

(DIS2)

Some of the students indicated that the work shifts established by the hospital were a challenge in ensuring continuous care. Faced with this situation, the nursing students noted that with these devices they used apps to record the patient’s clinical information, which could be accessed at any place or time of the day. Furthermore, the insecurity due to the lack of experience of the nursing students during their clinical practicums made the WhatsApp groups between peers or with preceptors a way for them to quickly obtain information to act in certain situations. These apps and WhatsApp groups were thought of as an essential way to share knowledge and guarantee continuous patient care.

“I saw how nurses would take pictures of wounds or ulcers and upload them to an app to see how they change.”

(DIS4)

“I’ve used WhatsApp groups for many things like resolving doubts about patient care, sharing experiences and opinions, supporting each other, it’s incredible… you immediately have an answer or at least alternatives…and it feels welcoming, you don’t feel as embarrassed and you ask more questions, for me it’s something positive.”

(FGS1)

#### 3.2.2. Empowering Clinical Work

The nursing students felt that smartphones helped them in the clinical setting by providing them with security and self-confidence to be able to independently manage and successfully resolve clinical situations that occurred during the workday, increasing their potential.

“Simply having my smartphone with me makes me feel safer and more relaxed. Sometimes I’ve needed to know something and the nurse wasn’t with me, thanks to my smartphone I was able to resolve it on my own.”

(DIS5)

The inconsistent actions of different nursing professionals when performing the same procedure led nursing students to question how they should correctly carry out the procedure. Smartphones allowed the nursing students to access clinical information in order to act appropriately when faced with the situation.

“I’ve seen a nurse do something one way and later another way, while in class they taught us to do it a different way. You don’t know which way is right…so you look it up on your smartphone and find out how to do it.”

(FGS2)

### 3.3. Impact of Smartphone Use on Patient Care

This topic analysed the experiences of the nursing students in regard to the impact of smartphone use on patient care practise. Included are the subtopics “Communication as the key link between nurse and patient”, “Compromising the clinical safety of the patient” and “The search for ethics: the need to change smartphone use in the professional environment.” 

#### 3.3.1. Communication as the Key Link between the Nurse and Patient

Communication was acknowledged by the participants as an essential factor to establish trust with the patient. The nursing students believed that using their smartphones in the presence of patients interfered with their communication. The nursing students perceived how this action provoked negative feelings in the patient and a loss of empathy on the part of the professionals, breaking the bond established between the nurse and the patient.

“One time my smartphone rang while I was in a patient’s room, interrupting our conversation. The patient was taken aback, as if he wasn’t my priority. I could tell that he felt neglected and his expression changed… as if I had disappointed him.”

Likewise, some participants recognised that receiving personal information through their smartphone while communicating with patients interfered with their attitude, resulting in the loss of relevant clinical information. Thus, smartphone use during communication with patients could negatively affect the image perceived of the professionals by the patients.

“I received bad news (by smartphone) while I was talking with a patient, which directly affected me. From that moment on, I didn’t have the same motivation or concentration, I didn’t pay attention to the conversation I was having with the patient, meanwhile he was telling me personal things that were important to him.”

(FGS2)

#### 3.3.2. Compromising the Clinical Safety of the Patient

The participants identified smartphones as a danger for patient security that interfere with the attention and quality of care given by not making the patient their first priority. The students considered smartphones to be a distraction when performing clinical interventions due to the continuous disruptions caused, as well as the inability to control the impulse to check their messages.

“I always bring my mobile phone with me to the clinical practicums. When I receive a WhatsApp, I want to quickly finish what I’m doing so that I can look at it, I can’t help it… it’s out of my control.”

(DIS2)

Some of the participants acknowledged having made mistakes as a result of improperly using their smartphones, compromising the patient’s health. Moreover, some students conceived this fact as “carelessness,” downplaying it without being aware of the consequences that it could entail, indicating a lack of responsibility.

“One day they sent me to administer a Nolotil and to do an electrocardiogram on a patient but because I was on WhatsApp, I did it to the patient in a different room. I mean, I went back to the room I was in before… it was a moment of carelessness.”

(DIS6)

The nursing students considered smartphones in the clinical setting to be a potential health hazard, especially during the current pandemic, due to the risk of infection or cross contamination.

“You’re always touching your smartphone, later you touch your face or you touch the patient, it’s a source of infection. Also, during the current pandemic, it is very dangerous.”

(FGS1)

#### 3.3.3. The Search for Ethics: The Need for Change of Smartphone Use in the Professional Environment

The participants perceived that in certain clinical situations, smartphone use could lead to a lack of professional ethics by compromising the privacy and confidentiality of the patient. The students acknowledged obtaining personal information, without the patient’s permission, and even sending the information to others.

“On some occasions I’ve seen something interesting when I’m with the patient, so I took pictures with my smartphone without asking the patient and even shared it with my workmates.”

(DIS4)

The students identified that the inappropriate use of smartphones could be solved by developing codes of conduct in order to improve aspects in the work environment and setting, such as performance and relationships with colleagues. Likewise, other participants commented that the improper use of smartphones in the clinical environment could affect the quality and public image of health institutions.

“I think measures should be taken and (smartphone) use should be regulated… also, it would be a positive aspect for teamwork.”

(DIS3)

“One day, a patient turned in a customer complaint form because the nurse used her mobile phone in the patient’s room… it was a big problem at the hospital.”

(DIS8)

Teaching nursing students about the use and possibilities offered by smartphones was another aspect they considered relevant in order to optimise the available resources of these devices. Furthermore, the training was perceived as a way to be able to safely use their smartphone in the clinical setting.

“When you arrive at the hospital, no one tells you how you should use your smartphone. You don’t know if you should put it on silence mode, have the volume on or turn it off. You don’t know where you can use it either. You feel lost.”

(DIS1)

## 4. Discussion

This study aimed to explore nursing students’ perceptions of their lived experiences of smartphone use in the clinical setting in regard to patient safety. There is little evidence on the ways in which these devices are used and the impact they may have in the clinical setting [13]. In regard to the results of this study, the nursing students reported using their smartphones in the clinical setting for personal reasons. Previous studies show that nursing professionals and students have increased their smartphone use for personal reasons in the clinical setting and that the main activity was using social networks [10,13]. These data coincide with that reported by the participants of this study, who emphasised their need to be constantly connected to social networks. This need to be connected could be due to a fear of missing out by not connecting to social networks [36]. In addition, the students reported feeling anxiety, as well as having recurring thoughts when they did not have their smartphones. These characteristics could be indicators of problematic smartphone use, or nomophobia [21,22]. On the other hand, smartphone use for personal reasons in the clinical setting could be a means of catharsis and an escape from reality, helping to minimise emotional stress. Although these positive aspects might not be considered essential in the clinical setting, they show the potential for social networks to improve the social–emotional well-being of nursing professionals [37]. However, the negative impact on relationships with colleagues can interfere with the work environment and contribute to lower levels of satisfaction in the clinical setting [38]. 

Smartphone use in the clinical setting could lead to a failure to fulfill the duties of the nursing professionals. Moreover, problematic smartphone use has been related to a decrease in clinical performance and delaying the main duties of professional nurses [18]. The nursing students reported that their preceptors did not train them properly due to their smartphone use. However, studies have not been found that explore how the smartphone use by preceptors in the clinical setting could interfere with the training and education of the nursing students. It is necessary to further investigate this aspect, as preceptors are role models for the nursing students and portray the image of a professional nurse. Similarly, what preceptors teach, see or do in the clinical setting will establish the idea of a professional nurse for the nursing students [39].

On the other hand, smartphones have been considered a support mechanism for patient care during their clinical practicums. Nevertheless, the quality of the information accessed via the internet tends to vary, and misinformation can easily be shared [40]. This area must be explored in greater depth, because the quality of sources consulted may be inadequate and the students may be looking up information directly on Google [41]. On the other hand, support groups were considered useful for their clinical work. Previous studies indicate that these groups can reduce medication errors, maintain continuity of care and enable clinical decisions to be made faster [42]. Likewise, smartphone use in the clinical setting allowed the nursing students to feel empowered to care for patients. In this sense, smartphones in the clinical setting may encourage clinical decision-making by increasing self-confidence and clinical efficacy, and reducing stress during the clinical practicums [5,8]. 

Regarding the impact of the use of smartphones on patient care, the participants expressed that it caused an interference in communication, as well as a loss of empathy towards the patient. In the clinical setting, interpersonal communication of the nursing professional with the patient is considered the basis of nursing care [43]. Similarly, it is essential for nursing students to communicate effectively to establish trust with the patient in order to improve adherence to treatment, patient satisfaction, quality of care, and the care provided [44,45]. Likewise, a decrease in empathy could lead to dehumanisation of care and an increased risk of harm to patient [46]. This decrease in empathy could be associated with the use of social networks, which has been shown to interfere with the ability to perceive the emotions of others [47]. Also, the use of social networks has worsened communication skills [48]. 

The results of this study showed that distractions and disruptions caused by smartphones could compromise patient safety. Along the same lines, several studies have shown that smartphone use can increase the risk of distraction in clinical practises [30,31], leading to more clinical errors and a loss of relevant clinical information [49]. On the other hand, students identified smartphone use in the clinical setting as a potential risk of infection, particularly during the current pandemic. Likewise, other studies affirmed that the improper use of smartphones can increase the risk of transmitting bacteriological agents to patients, increasing hospital infections [17].

The loss of confidentiality and privacy of patients due to the improper use of smartphones was another aspect that the nursing students reported. Therefore, nursing students and professionals should be aware of how this lack of ethics could negatively affect their professional image and the nursing profession [15]. Thus, the need to provide further education on the appropriate use of smartphones in the clinical setting to students and nursing professionals is essential in order to develop safe, professional and ethical practises. Along the same lines, inappropriate use of smartphones could be solved by developing codes of conduct for smartphone use in the clinical setting, as well as with training on how to use these devices properly. Additionally, other studies have indicated that nursing students may have the ability to use these devices to communicate but may not have the skills to use them in a professional way in the clinical setting [50,51]. Therefore, it would be necessary to integrate this training into the nursing curricula in order to educate students on the resources and uses of smartphones to promote and incorporate them correctly in the clinical setting. 

This study should be interpreted considering a number of limitations. First, the selection of nursing students was carried out in only one faculty of nursing studies. Also, the sample size was small and biased towards nursing students’ experiences and perceptions of smartphone use in the clinical setting. It should also be taken into account that the duration of data collection techniques was shorter than recommended. Finally, the formulation of questions at three levels of analysis, descriptive, structural and contrast, was not considered. However, the sample was representative of the cohorts of modern-day students and provided insightful data. Similarly, future research could incorporate a broader representation of participants in order to obtain greater diversity in the experience of the participants and greater confidence in the transferability of the findings. 

## 5. Conclusions

The nursing students used their smartphones in the clinical setting for personal reasons, reporting that they felt the need to be connected and escape from the clinical environment. Smartphones have been considered a useful tool for improving clinical decision-making. However, smartphone use in the clinical setting may worsen the work environment as well as the learning of nursing students. In addition, improper smartphone use could interfere with patient care, impairing communication between the nursing professional and the patient. Furthermore, smartphones could constitute a threat to the privacy, confidentiality, and clinical safety of the patient. For this reason, it would be necessary to develop educational programmes to educate nursing students on the uses of smartphones in the clinical setting to optimise this resource and also to establish codes of conduct for smartphone use in the clinical setting.

## Figures and Tables

**Table 1:** Guide used for the semi-structured interview.

Interview Guide Questions
What is your experience using the smartphone during clinical practices?
Could you tell me in which situations you use the smartphone in the clinical practices? Why?
What is your opinion on the use of the smartphone in the clinical practices?
How do you think the use of a smartphone could interfere in your clinical training?
Tell me about your experience in the patient care process and using the smartphone
How do you think that the use of the smartphone could interfere in the relationship between professionals or nursing students?
Tell me about situations that you have witnessed in which the smartphone has been used in the presence of the patient
Is there anything else you would like to say about this theme?

**Table 2:** Sociodemographic data of the participants (N = 24).

Participant	Age (Year)	Gender	Course	Practicums Performed
FGS1-1	22	M	4	5
FGS1-2	31	F	2	1
FGS1-3	19	F	2	1
FGS1-4	23	F	4	6
FGS1-5	19	F	2	1
FGS1-6	19	F	2	1
FGS1-7	32	F	3	3
FGS2-1	19	F	2	1
FGS2-2	26	F	4	6
FGS2-3	19	M	2	1
FGS2-4	21	M	3	3
FGS2-5	19	F	2	1
FGS2-6	19	F	2	1
FGS2-7	19	F	2	1
DIS1	19	M	2	1
DIS2	20	F	3	3
DIS3	19	F	2	1
DIS4	24	F	4	6
DIS5	23	M	4	6
DIS6	19	F	2	1
DIS7	21	F	3	3
DIS8	19	F	2	1
DIS9	22	F	4	6
DIS10	20	F	2	1

FGS = focus group smartphone; DIS = in-depth interview Smartphone.

**Table 3:** Units of meaning, subthemes and main themes of the analysis.

Units of Meaning	Sub-Themes	Main Themes
Satisfaction of personal needs, need to be connected, catharsis, work isolation	Smartphones as a means of personal gratification	Theme 1. Smartphone use as a personal resource in the clinical setting
Displacement of main tasks, labor non-compliance, poor training	Failure of professional competence	
Consult medication, visualize clinical procedures, record clinical information, support groups	Support mechanism for care	Theme 2. Smartphone as a support mechanism for making clinical decisions
Self-confidence, problem solving, critical thinking	Empowering clinical work	
Patient communication, lack of empathy, loss of relevant information	Communication as the key link between the nurse and patient	Theme 3. Impact of smartphone use on patient care
Patient care, quality of care, distraction, medication errors, risk of infection	Compromising the clinical safety of the patient	
Threat of privacy and confidentiality, establish codes of conduct, quality indicator, development of educational programs	The search for ethics: the need for change of smartphone use in the professional environment	

## Data Availability

Data available on request due to restrictions.
